# Determination of Morphine and Codeine in Human Urine by Gas Chromatography-Mass Spectrometry

**DOI:** 10.1155/2013/151934

**Published:** 2013-10-10

**Authors:** Xiaoqian Zhang, Mengchun Chen, Gaozhong Cao, Guoxin Hu

**Affiliations:** ^1^School of Pharmacy of Wenzhou Medical University, Wenzhou 325035, China; ^2^The First Affiliated Hospital of Wenzhou Medical University, Wenzhou 325000, China

## Abstract

A sensitive and selective gas chromatography-mass spectrometry (GC-MS) method was developed and validated for the determination of morphine and codeine in human urine. The GC-MS conditions were developed. The analysis was carried out on a HP-1MS column (30 m × 0.25 mm, 0.25 **μ**m) with temperature programming, and Helium was used as the carrier gas with a flow rate of 1.0 mL/min. Selected ion monitoring (SIM) mode was used to quantify morphine and codeine. The derivation solvent, temperature, and time were optimized. A mixed solvent of propionic anhydride and pyridine (5 : 2) was finally used for the derivation at 80°C for 3 min. Linear calibration curves were obtained in the concentration range of 25–2000.0 ng/mL, with a lower limit of quantification of 25 ng/mL. The intra- and interday precision (RSD) values were below 13%, and the accuracy was in the range 87.2–108.5%. This developed method was successfully used for the determination of morphine and codeine in human urine for forensic identification study.

## 1. Introduction

Morphine and codeine are naturally occurring alkaloids in opioid plants, have long been used as a drug, and are also abused. While the presence of illicit drugs or their metabolites in urine is an evidence of intake, their concentrations in blood are expected to correlate with their effects on the central nervous system [1]. Morphine is a powerful narcotic analgesic and highly addictive. Codeine is a potent *μ*-opioid receptor agonist which is used for the treatment of adult cough. Simultaneously, there have been athletes in sports competitions who use a larger dose in order to improve performance. This practice is contrary to the principle of fair competition and also harmful to the health of the athletes' body. Heroin as one of the most widely abused drug, rapidly metabolized to 6-monoacetylmorphine (6-MAM) once inside the human body. This specific heroin metabolite 6-MAM is detected at a higher concentration usually within 2 to 4 hours, and after six hours, has not been detected in the urine. The absence of 6-MAM in urine, however, morphine is both a well-known pharmaceutical agent and an important metabolite of codeine and heroin which have relatively long a detection time. Morphine and codeine analysis of urine is used in forensic toxicology to study drug addiction.

There are numerous papers published about the simultaneous determination of Morphine and Codeine in human fluids, including the micellar electrokinetic chromatography (MEKC) method [2], disposable pipette extraction (DPX) method [3], high performance liquid chromatography method [4], liquid chromatography-mass spectrometry [5], and liquid chromatography/triple quadrupole tandem mass spectrometry (LC/MS/MS) method [6–8]. Several gas chromatography-mass spectrometry (GC-MS) methods have been developed for the analysis of codeine, morphine, or other opiates. Much attention has been directed to the confirmation of morphine and codeine in urine by GC-MS [9]. A few methods have been developed specifically for the analysis of 6-acetylmorphine (6-AM) with morphine and codeine because all three drugs are often present after heroin use. Assays of morphine and codeine by GC-MS are capable of high sensitivity, specificity, and selectivity. GC-MS is superior to other analytical methods which provide important diagnostic value to study the drug abuse. The aim of this study was to establish methods and seek out more reliable identification and quantitation of morphine and codeine for detection addicts sample.

Currently, urine sampling has been extensively employed for the evaluation of drug consumption. Although through in saliva is another approach; the reliability of saliva analysis is limited by the fact that analyte levels, and even the availability of required sample volume, are again dependent on several physiological factors, nutrition and fluid intake, while the biological effects of the consumed illicit substance may also be a significant factor [10]. The identification of chronic consumers or the late verification of a single intake is feasible using hair as a matrix [11], but it is not suitable for the early verification of consumption. Urine is a preferable matrix for analytical purposes in comparison with saliva because of the minimal discomfort caused to sampled individuals, so it is widely available.

Sample preparation is a key step for the determination of drugs in biological samples. The simple and effective ethyl acetate extraction was employed in our work, and ethyl acetate was adopted because of its high extraction efficiency. Pyridine is a catalytic solvent for reactions with propionic anhydride. Propionic anhydride was chosen as the derivatization reagent because it exhibited better effect than acetic anhydride or trifluoroacetic acid anhydride, which could provide preferable stability, and the disadvantage of acetyl derivatives indistinguishable from morphine and the 6-AM can be avoided. Kushnir et al. [12] evaluated propionic anhydride, MBTFA, HFAA, and BSTFA for GC-MS analysis of 6-AM. They concluded that propionic anhydride gave accurate, precise, and sensitive results while providing compatibility with other methods on the same GC-MS instrument. Residual derivatization reagent in the injector will react with drugs in other methods not intended for derivatization. The derivatization procedure accommodates the analysis of opioids commonly requiring GC-MS confirmation in urine. Difficulties were expected to arise due to a number of reasons. Concentrations of the analytes in the samples were expected to be smaller than the low end of the therapeutic range (25 ng/mL), which highlighted the importance of efforts aimed at increasing the sensitivity of detection. Validation of the analytical method also posed certain requirements. The relative standard deviation of the retention parameters of the target compound was required not to exceed 5% relative standard deviation.

## 2. Experimental

### 2.1. Chemicals and Reagents

Morphine [10 *μ*g/mL in methanol] and codeine [10 *μ*g/mL in methanol] solutions were obtained from the Institute of Forensic Science under the Ministry of Justice (Shanghai, China). Sodium hydroxide (purity >98.0%) was purchased from Sigma-Aldrich Trading Co (Shanghai, P.R., China), and ethyl acetate (purity >98.0%) was purchased from Siyou Chemical Reagent Co., Ltd (Tianjin, China), and propionic acid anhydride (purity >98.0%) was purchased from Sinopharm Chemical Reagent Co., Ltd (Beijing, China). Pyridine was from Shenbo Chemical Co., Ltd (Shanghai, China). While methanol was obtained from Siyou Chemical Reagent Co., Ltd (Tianjin, China). Ultrapure water was prepared by a Milli-Q purification system from Millipore (Bedford, USA). All other chemicals were analytical pure and used without further purification.

### 2.2. Instrumentation and Conditions

Analysis was performed on an Agilent 6890N gas chromatograph (GC) coupled with an Agilent 5975B mass spectrometer (MS, Agilent Technologies, Wilmington, DE, USA). Samples were injected using an Agilent autosampler unit.

**Figure 1:**
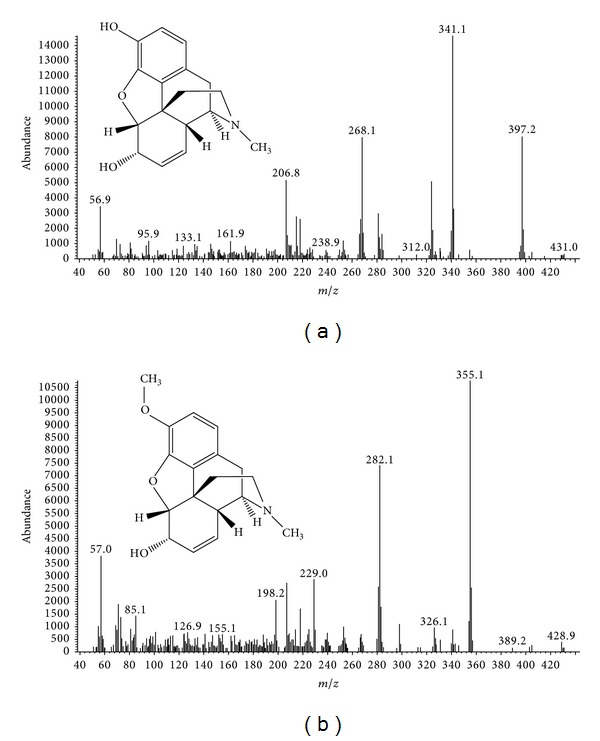
Mass spectra of morphine (a) and codeine (b) in SIM mode with EI (+) source.

The capillary column used was a HP-1MS [30 m × 0.25 mm, 0.25 *μ*m]. Helium was the carrier gas at a flow rate of 1.0 mL/min. The temperature program was: initial temperature, 100°C for 1.5 min; ramp at 25°C/min to 280°C and held for 15 min; injection temperature, 250°C; and transfer line, 280°C. Sample injection volume was 1 *μ*L. Splitless injection mode was used. Electron impact ionization was performed at 70 eV energy and at a 230°C ion source temperature. The quadrupole temperature was 150°C. The MS was operated in single ion monitoring (SIM) mode. SIM mode was applied to quantify analyzes using target ions at *m*/*z* 341, 397, and 268 for morphine propionyl compound and *m*/*z* 229, 355, and 282 for codeine propionyl compound (Figure 1).

### 2.3. Sample Preparation

The primary standard stock solutions of morphine (100 *μ*g/mL) and codeine (100 *μ*g/mL) were separately prepared in 10 mL volumetric flasks with urine; 10% NaOH was added dropwise until pH 9.0–9.2 was reached, and 1.0 mL of borax buffer solution was added. To this, 3 mL of extraction solvent (ethyl acetate) was added and vortex-mixed on a vortexer for 2.0 min, followed by centrifugation at 3000 r/min for 5 min. The supernatant organic layer was transferred into a 5 mL glass test tube and dried under air stream at 60°C. The dried residue was reconstituted in 50 *μ*L of propionic anhydride and 20 *μ*L of pyridine. All reagents were vortex-mixed, then heating for 3 min at 80°C and dried under air stream at 60°C. The dried residue was reconstituted in 50 *μ*L of methanol, and 1 *μ*L of this solution was injected into GC-MS.

### 2.4. Method Validation

Specificity was determined by analysis of blank urine, without addition of morphine and codeine to determine possible interference with these compounds.

To evaluate the linearity, the calibration curves were generated using the analyte peak area by linear regression on three consecutive days. The LLOQ was estimated in the process of calibration curve construction and was defined as the lowest concentration for which precision (RSD) was better than 20%.

QC samples at three concentration levels (50, 200, and 1600 ng/mL for morphine and codeine) were analyzed to assess the accuracy and precision of the method. Again, the assays were performed on three separate days, and on each day six replicates of the QC samples at each concentration level were analyzed. The assay accuracy was calculated as relative error. The assay precision for each QC level was determined as the relative standard deviation (RSD) of the measured concentrations. The intra- and interday precisions were required to be below 15%, and the accuracy was required to be within ±15%.

Stability in urine was assessed in the autosampler at room temperature for 12 h. The effect of three freeze-thaw cycles was also investigated.

## 3. Results and Discussion

### 3.1. Selectivity and Linearity

**Figure 2:**
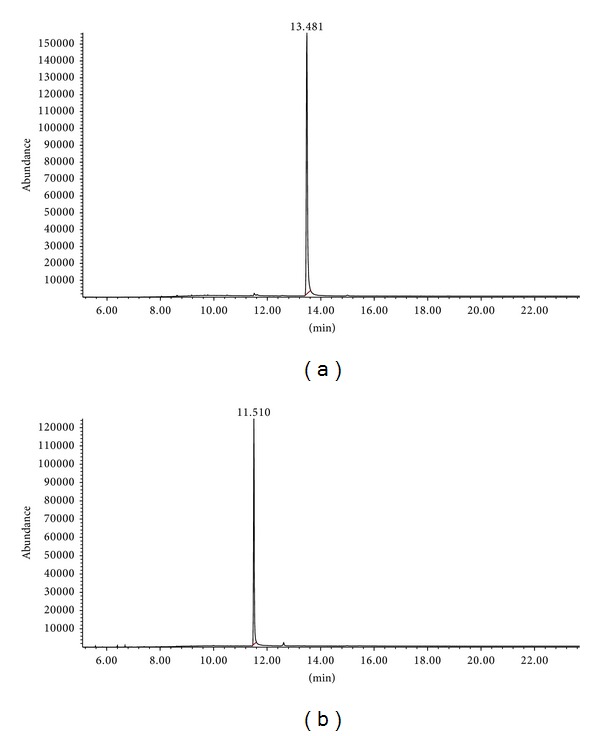
Chromatograph of urine sample containing 1600 ng/mL morphine (a) and codeine (b) processed through the procedure.

Figure 2 shows the typical chromatograms of a blank urine sample spiked with morphine and codeine. No interfering endogenous substances were observed at the retention times of the morphine and codeine.

Calibration curves for morphine and codeine were generated by linear regression of peak area ratios against concentrations, respectively. The regression equation for the calibration plot were *Y* = 2270.9*C* + 202.3 with *r* = 0.9974 for morphine, and *Y* = 3099.0*C* + 31625.7 with *r* = 0.9958 for codeine (*Y* is the peak area of analyte, and *C* is the concentration of analyte in human urine), and concentrations are in the range 25–2000 ng/mL for morphine and codeine, respectively.

The LLOQ for morphine in human urine was 25 ng/mL and the precision and accuracy at LLOQ were 10.5% and 87.6%, respectively. The LLOQ for codeine in human urine was 25 ng/mL and the precision and accuracy at LLOQ were 13.8% and 88.9%, respectively. 

### 3.2. Precision, Accuracy, and Extraction Recovery

The precision of the method was determined by calculating RSD for QCs at three concentration levels over three validation days. Intraday precision was 12% or less and the interday precision was 13% or less at each QC level. The accuracy of the method ranged from 87.2% to 99.7% at each QC level. Assay performance data are presented in Table 1. The aforementioned results demonstrate that the values are within the acceptable range and the method is accurate and precise. The recovery of morphine and codeine was evaluated by comparing peak area ratios of extracted QC samples with those of reference QC solutions reconstituted in blank urine extracts. Mean recoveries of morphine and codeine were better than 75.5%.

### 3.3. Stability

All the stability studies of morphine and codeine in human urine were conducted at three concentration levels (50, 200, and 1600 ng/mL for morphine and codeine) with three replicates for each concentration. The stability results showed that morphine and codeine in human urine were stable during three freeze-thaw cycles. Stability of morphine and codeine extracts in the sample solvent on autosampler was also observed over a 12 h period. The results of stability experiments are listed in Table 2.

## 4. Conclusions

A stable, selective, and sensitive GC-MS method has been developed for the simultaneous determination of codeine and its metabolite morphine in human urine. This developed method with derivatization for sample preparation was successfully applied for the determination of morphine and codeine in human urine for methodological study.

## Figures and Tables

**Table 1:** The results of precision, accuracy, and recovery studies for morphine and codeine in human urine (*n* = 6).

Compound	Concentration	RSD (%)		RE (%)		Recovery (%)
	(ng/mL)	Intraday	Interday	Intraday	Interday	
Morphine	50	4.3	8.2	−0.3	−6.5	86.1
	200	4.4	7.4	−5.2	−10.1	75.5
	1600	5.3	9.3	−6.9	5.7	80.2

Codeine	50	10.5	13.0	8.5	−7.3	78.9
	200	11.4	9.8	−3.9	−12.8	79.7
	1600	5.2	12.9	−9.9	−8.2	86.8

**Table 2:** Summary of stability of morphine and codeine under various storage conditions (*n* = 3).

Compound	Condition	Concentration (ng/mL)	RSD	RE
		Added	(%)	(%)
Morphine	Three freeze–thaw cycles	50	2.5	−10.1
		200	1.4	−8.9
		1600	4.3	−12.5
	Autosampler ambient 12 h	50	2.1	−10.5
		200	1.3	−6.2
		1600	3.5	−9.2

Codeine	Three freeze–thaw cycles	50	3.2	−8.9
		200	3.3	−11.2
		1600	3.0	−14.1
	Autosampler ambient 12 h	50	3.3	−11.1
		200	3.2	−12.9
		1600	3.3	−14.4
